# The decapping activator Edc3 and the Q/N-rich domain of Lsm4 function together to enhance mRNA stability and alter mRNA decay pathway dependence in *Saccharomyces cerevisiae*

**DOI:** 10.1242/bio.020487

**Published:** 2016-08-19

**Authors:** Susanne Huch, Maren Müller, Mridula Muppavarapu, Jessie Gommlich, Vidya Balagopal, Tracy Nissan

**Affiliations:** Department of Molecular Biology, Umeå University, Umeå SE-901 87, Sweden

**Keywords:** P bodies, Deadenylation, Exosome, mRNA decapping, mRNA decay, mRNA stability

## Abstract

The rate and regulation of mRNA decay are major elements in the proper control of gene expression. Edc3 and Lsm4 are two decapping activator proteins that have previously been shown to function in the assembly of RNA granules termed P bodies. Here, we show that deletion of *edc3*, when combined with a removal of the glutamine/asparagine rich region of Lsm4 (*edc3*Δ *lsm4*Δ*C*) reduces mRNA stability and alters pathways of mRNA degradation. Multiple tested mRNAs exhibited reduced stability in the *edc3*Δ *lsm4*Δ*C* mutant. The destabilization was linked to an increased dependence on Ccr4-mediated deadenylation and mRNA decapping. Unlike characterized mutations in decapping factors that either are neutral or are able to stabilize mRNA, the combined *edc3*Δ *lsm4*Δ*C* mutant reduced mRNA stability. We characterized the growth and activity of the major mRNA decay systems and translation in double mutant and wild-type yeast. In the *edc3*Δ *lsm4*Δ*C* mutant, we observed alterations in the levels of specific mRNA decay factors as well as nuclear accumulation of the catalytic subunit of the decapping enzyme Dcp2. Hence, we suggest that the effects on mRNA stability in the *edc3*Δ *lsm4*Δ*C* mutant may originate from mRNA decay protein abundance or changes in mRNPs, or alternatively may imply a role for P bodies in mRNA stabilization.

## Abstract (precis)

**Summary:** A strain mutated in two decapping activators, previously implicated in P body assembly, has reduced mRNA stability and increased dependence on decapping and Ccr4-dependent deadenylation for mRNA degradation.

## INTRODUCTION

The degradation of mRNAs is a fundamental process in the control and modulation of gene expression (Pérez-Ortín et al., 2012). The mRNA degradation process commences with shortening of the poly(A) tail by the Ccr4/Not and Pan2/3 deadenylation complexes. In yeast and other eukaryotes, after mRNA is deadenylated, further decay can occur through one of two pathways: the 5′-to-3′ pathway, which is dependent on removal of the m7G cap from mRNA, and the 3′-to-5′ pathway, in which degradation occurs via the action of the exosome complex (Parker, 2012). Whereas one pathway can partially substitute for the absence of the other, in yeast most mRNAs are degraded by the decapping-dependent pathway (Beelman et al., 1996; Cao and Parker, 2001; Haimovich et al., 2013; Medina et al., 2014; van Dijk et al., 2011). This degradation pathway begins with the removal of the m7G cap from the mRNA by the decapping complex composed of Dcp1 and its catalytic subunit, Dcp2, which is followed by 5′-to-3′ exonucleolytic digestion via Xrn1; however, mRNA cap removal occurs at a slower rate in the absence of accessory proteins, termed mRNA decapping activators (Borja et al., 2011; Fromm et al., 2012; Nissan et al., 2010; Parker, 2012; Valkov et al., 2016). Most prominent among these factors are Dhh1, Pat1, Edc3 and the Lsm1-7 complex.

Edc3 is unusual among the decapping activators in that its absence has only a minor effect on general mRNA stability in yeast. The *edc3* deletion does not affect mRNA abundance genome-wide, with the exception of the *RPS28B* and *YRA1* mRNAs (Badis et al., 2004; Dong et al., 2007; Sun et al., 2013). In addition, in yeast, the absence of Edc3 does not affect the mRNA decapping and stability of individual mRNAs or the mRNA half-lives determined genome-wide (Decker et al., 2007; Kshirsagar and Parker, 2004; Parker, 2012; Sun et al., 2013). However, Edc3 has an additional structural role in the assembly of cytoplasmic foci of mRNA and proteins involved in mRNA degradation in other contexts (Decker et al., 2007; Teixeira and Parker, 2007). These structures, termed cytoplasmic processing bodies (P bodies), contain the Ccr4/Not and Pan2/3 deadenylase complexes in addition to decapping factors (Bett et al., 2013; Cougot et al., 2004; Teixeira and Parker, 2007). Cytoplasmic mRNA decay is thought to occur within P bodies since they contain decaying mRNA (Sheth and Parker, 2003).

The *edc3*Δ mutant's structural role results in the absence of microscopically observable P bodies only without aeration or in respiratory deficient yeast (Decker et al., 2007). To eliminate microscopically visible P bodies, the removal of the prion-like glutamine/asparagine rich domain of the Lsm4 protein is also required. Lsm4 is an essential protein that is a component of both the decapping activating Lsm1-7 complex and the Lsm2-8 complex, which is part of the U6 snRNP (Beggs, 2005; Tharun, 2009). Whereas depletion of the Lsm4 complex inhibits mRNA decapping (Tharun et al., 2000), the absence of the Q/N rich C-terminus of Lsm4 (*lsm4*Δ*C*) has been shown to cause a slight increase in mRNA half-lives (Decker et al., 2007; Reijns et al., 2008).

The *edc3*Δ and *lsm4*Δ*C* double mutant has been used by multiple groups to examine the role of P bodies in mRNA stability and additional cellular functions (Aronov et al., 2015; Decker et al., 2007; Lavut and Raveh, 2012; Simpson et al., 2014). In this study, we sought to characterize the effect of the *edc3*Δ *lsm4*Δ*C* mutant on mRNA metabolism by using yeast as a simple eukaryotic model that is tractable to multiple types of experimental manipulation. When the mutations were combined in the *edc3*Δ *lsm4*Δ*C* strain, we found that multiple mRNAs were reduced in stability, whereas the stability of other mRNAs was unaffected. To further examine the effect of the *edc3*Δ *lsm4*Δ*C* mutant on the mRNA stability, we used mutants in which the major pathways of mRNA degradation were compromised. Our results suggest that the *edc3*Δ *lsm4*Δ*C* mutant has a faster degradation rate, owing to greater reliance on deadenylation by the Ccr4/Not complex as well as mRNA decapping. We also provide evidence that links the *edc3*Δ *lsm4*Δ*C* mutant to altered mRNA translation and the subcellular localization of the mRNA decapping enzyme. Finally, we report that the *edc3*Δ *lsm4*Δ*C* mutation confers a survival advantage when yeast cells are exposed to long-term starvation.

## RESULTS

### mRNAs are destabilized by a synergistic effect of deletion of both EDC3 and the glutamine/asparagine rich domain of Lsm4

**Fig. 1.**
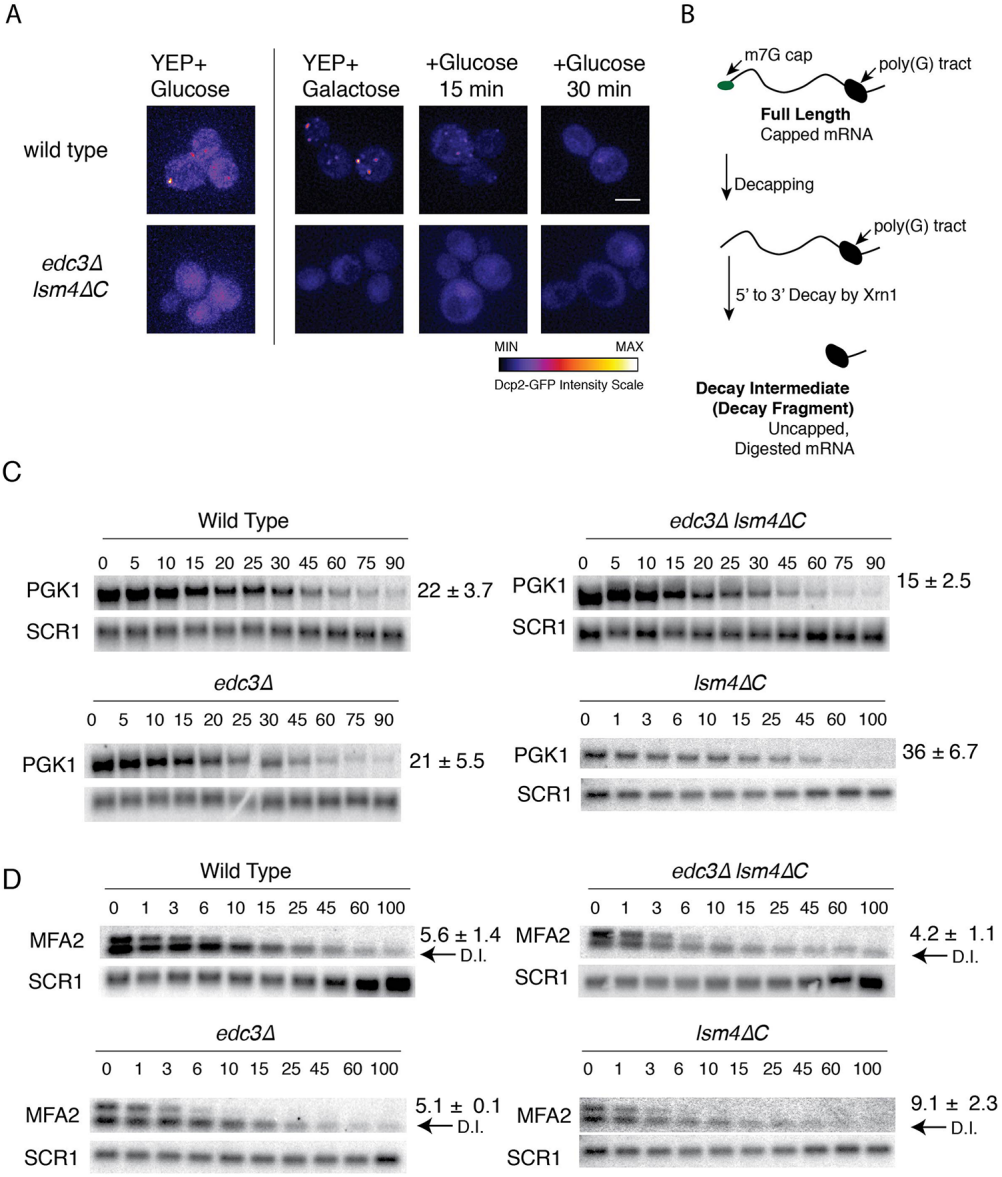
***PGK1* and *MFA2* mRNA stability in the *edc3*Δ *lsm4*Δ*C* mutant.** (A) Wild type and *edc3*Δ *lsm4*Δ*C* mutant yeast cells expressing Dcp2-GFP from its endogenous locus grown in SC+2% glucose or 2% galactose as indicated. Later time points depict the cells grown in galactose after being washed, resuspended, and grown in medium containing glucose. (B) Depiction of the full length capped mRNA and the decay fragment generated by decapping and 5′ to 3′ degradation, which was ultimately blocked by a poly(G) tract inserted into the 3′ UTR. (C) Northern blots for the half-life determination of *PGK1* mRNA in strains indicated. Time points (min) after transcriptional shut-off by glucose addition are shown. *SCR1* is the loading control. Error=s.d., *n*=3-15 biological replicates. (D) As above for *MFA2* mRNA, the half-life indicated to the right of the blots. Arrows labeled D.I. indicate the mRNA decay intermediate generated by decapping and 5′-to-3′ degradation blocked by the poly(G) tract in the 3′ UTR. Time points (min) are shown above the respective northern blots.

The *edc3*Δ *lsm4*ΔC mutant has been reported to be defective in the formation of P bodies (Decker et al., 2007). To confirm this result, we examined wild-type yeast and the *edc3*Δ *lsm4*ΔC mutant for the ability to form P bodies under unstressed growth. We defined P bodies as foci of the decapping enzyme, Dcp2-GFP. We observed P bodies in the wild-type strain when grown in glucose, but not in the *edc3*Δ *lsm4*Δ*C* mutant (Fig. 1A). The amount of P bodies was not consistently visible in all cells, as reported previously (Lui et al., 2014); however, the number of P bodies was consistently qualitatively greater in cells grown in galactose (Fig. 1A), whereas P bodies were absent from the *edc3*Δ *lsm4*Δ*C* mutant (Fig. 1A).

**Fig. 2.**
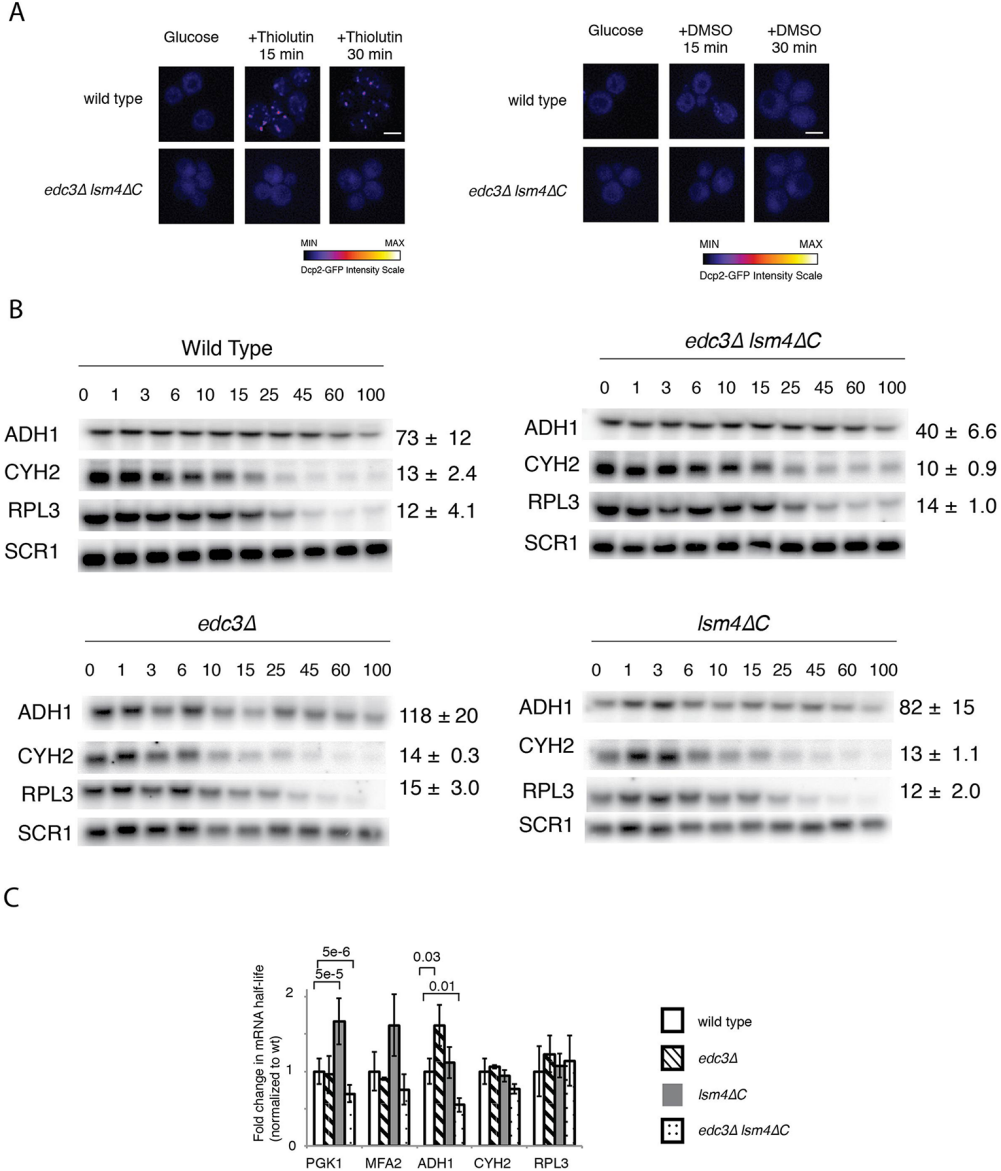
**Multiple mRNAs are destabilized in the *edc3*Δ *lsm4*Δ*C* mutant.** (A) Left: wild type and *edc3*Δ *lsm4*Δ*C* mutant yeast cells expressing Dcp2-GFP from its endogenous locus grown in SC+2% glucose after transcription was inhibited by thiolutin. Right: cells treated with DMSO alone. The images were plotted to the same contrast range and the scale bars represent 3 µm. Intensity of the GFP signal is indicated below. (B) Northern blots for *ADH1*, *CYH2*/*RPL28* and *RPL3* mRNA performed after thiolutin transcriptional shut-off. Error=s.d., *n*=3 biological replicates. Quantification of mRNA half-lives for mRNA normalized to *SCR1* as a loading control. Time points (min) are shown above the respective northern blots. (C) Quantification of mRNA half-lives for mRNA normalized to wild type half-life for each mRNA for the mRNA indicated. Statistically significant pairings according to a two-tailed *t*-test indicated with their *P* values.

We next determined the stability of mRNAs in the wild-type *edc3*Δ *lsm4*Δ*C* strain and in the individual *edc3*Δ and *lsm4*ΔC mutants. We examined *PGK1* and *MFA2* mRNAs, which have well-characterized mechanisms of decay (Cao and Parker, 2001) and have been found to be localized to P bodies in multiple studies (Aronov et al., 2015; Brengues et al., 2005; Decker et al., 2007; Sheth and Parker, 2003; Simpson et al., 2014; Teixeira et al., 2005; Zid and O'Shea, 2014). *PGK1* and *MFA2* mRNA were engineered with poly(G) tracts in their 3′ UTRs, thus allowing for the generation of mRNA decay intermediates after the mRNA is decapped and degraded 5′-to-3′ by Xrn1 (Fig. 1B) (Decker and Parker, 1993). This decay intermediate is more stable than the full-length mRNA, because further degradation of this intermediate occurs primarily from the 3′ end by the exosome (Fig. 1C, the decay intermediate is indicated by the arrow labeled D.I.) (Anderson and Parker, 1998). The *MFA2* and *PGK1* mRNAs were placed under the control of the genomically integrated *GAL1* promoter at the *CUP1* locus (Hatfield et al., 1996). Transcription was induced by growth in galactose. Half-lives were determined after inhibition of transcription with the addition of glucose. *PGK1* and *MFA2* represent typical stable and unstable mRNAs. To provide another example of a longer-lived mRNA, we also examined *ADH1* mRNA, which encodes an essential glycolytic enzyme. For shorter-lived mRNAs, we chose mRNAs encoding ribosomal proteins: *CYH2/RPL28* and *RPL3*. We chose mRNAs encoding for ribosomal proteins additional mRNAs because several studies have indicated that they behave differently than other mRNAs (Arribere et al., 2011; Gasch et al., 2000). The *ADH1*, *CYH2/RPL28* and *RPL3* mRNA half-lives were determined after inhibition of transcription with the drug thiolutin. We observed that thiolutin, compared with a DMSO control, induced P body formation in the wild-type yeast, but not in the *edc3*Δ *lsm4*ΔC mutant (Fig. 2A).

The mRNA half-lives varied from 5 to 73 min in the wild-type strain (Figs 1C,D and 2B). We found that the *PGK1* mRNA had a 46% shorter half-life in the *edc3*Δ *lsm4*Δ*C* mutant than in wild-type yeast (Fig. 1C), whereas *MFA2*'s half-life, although shorter, was not significantly altered (Fig. 1D). We found similar trends after using thiolutin to inhibit transcription. The *edc3*Δ *lsm4*Δ*C* mutant had shorter mRNA half-lives by at least 1.4-fold, except for those mRNAs encoding the ribosomal protein genes *CYH2/RPL28* and *RPL3* (Fig. 2B), results which are consistent with this class of mRNAs behaving differently in decay from bulk mRNA (Arribere et al., 2011; Gasch et al., 2000). None of the individual mutants exhibited such an effect. The *edc3*Δ strain had half-lives similar to those expected, with the exception of the *ADH1* mRNA (Decker et al., 2007; Kshirsagar and Parker, 2004). The *ADH1* half-life was instead significantly longer, thus suggesting inhibition of decapping as observed for other mRNAs with the *EDC3* decapping factor deletion (Dong et al., 2007; Sun et al., 2013). Half-lives in the *lsm4*Δ*C* background were similar to those of the wild-type or were modestly increased, as observed by other groups (Decker et al., 2007; Reijns et al., 2008), notably for the *PGK1* mRNA. Together, these data suggest that mRNAs tend to be destabilized in the *edc3*Δ *lsm4*Δ*C* mutant. Finally, reduced mRNA stability in the *edc3*Δ *lsm4*Δ*C* mutant was not due to growth defects, because the mutant grew similarly to wild-type yeast under optimal growth conditions (Fig. S1), as observed previously (Aronov et al., 2015; Decker et al., 2007).

### Reduction of mRNA stability in the *edc3Δ lsm4ΔC* mutant is consistent with increased deadenylation

To investigate the mechanism regulating mRNA stability in the *edc3*Δ *lsm4*Δ*C* mutant, we examined strains in which genes encoding proteins required for the different pathways of mRNA decay had been deleted, and we compared the results with those for the wild type and *edc3*Δ *lsm4*Δ*C* mutant. We used mutants defective in deadenylation (*ccr4*Δ) as well as decapping-dependent (*xrn1*Δ) and exosome-mediated (*ski2*Δ) mRNA degradation (Anderson and Parker, 1998; Parker, 2012; Tucker et al., 2002).

Mutations affecting a decay pathway required for more rapid decay in the mutant should result in greater relative mRNA stability. In yeast, for example, decapping dependent 5′-to-3′ decay is the primary pathway of mRNA degradation. When the cytoplasmic 5′-to-3′ exonuclease *XRN1* is deleted, mRNAs become more stable than when the cytoplasmic exosome (3′-to-5′ exonuclease) is inactivated, such as with a *ski2* deletion (Cao and Parker, 2001; Parker, 2012).

**Fig. 3.**
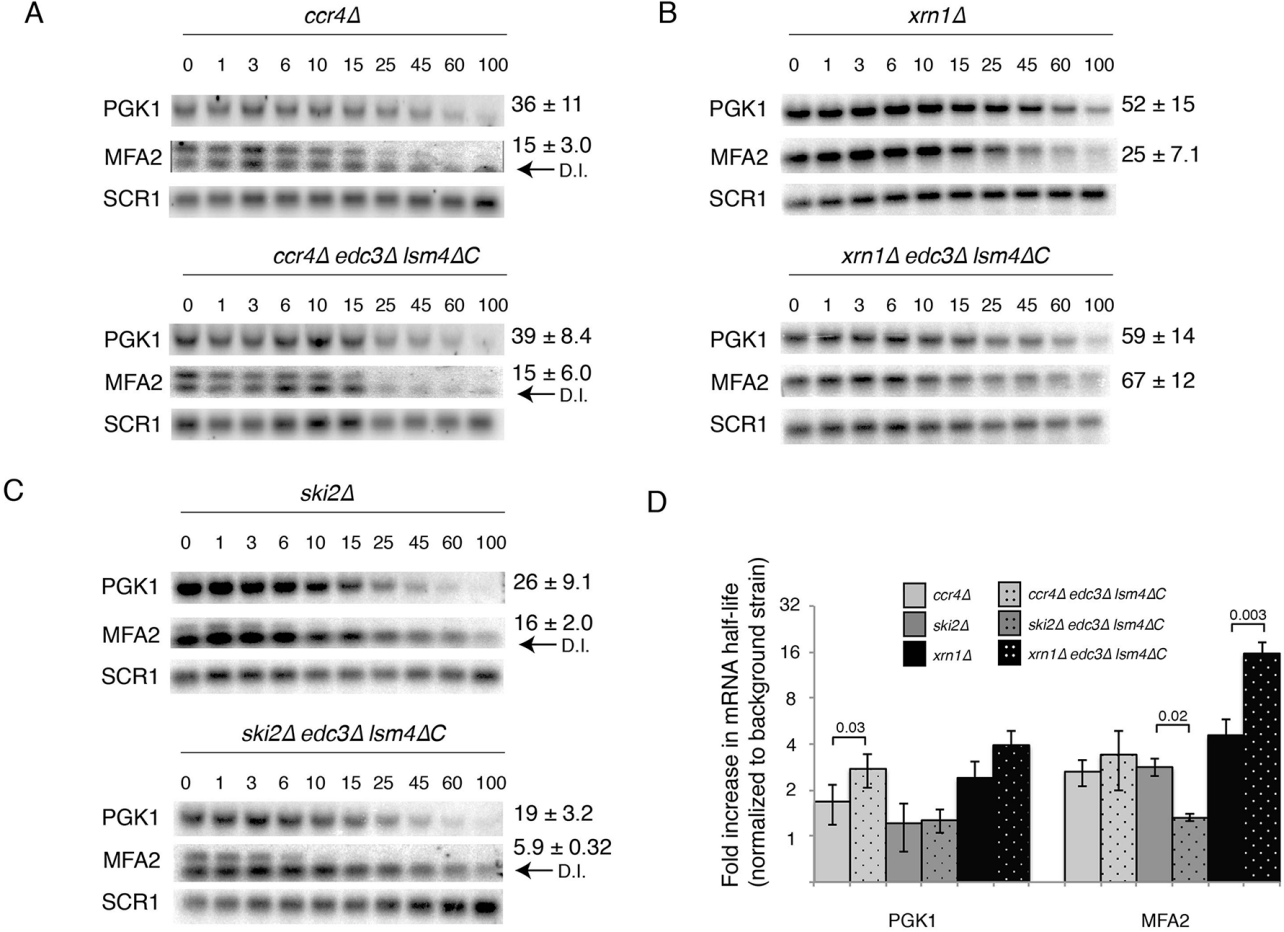
**Destabilization of mRNA in the *edc3*Δ *lsm4*Δ*C* mutant is attributable to increased deadenylation and decapping-dependent degradation.** (A) A *CCR4* deletion mutant in the wild-type and *edc3*Δ *lsm4*Δ*C* mutant backgrounds expressing *PGK1* and *MFA2* mRNA under the control of the *GAL* promoter when grown in YEP+galactose. Time points (min) indicated are after transcriptional shut-off by addition of glucose. The loading control is *SCR1*. Error=s.d.; *n*=5-6 biological replicates. (B) As above, but with an *xrn1*Δ mutation, *PGK1 n*=2-3 biological replicates, *MFA2 n*=3 biological replicates. (C) As above, but with a *ski2*Δ mutation, *PGK1 n*=5 biological replicates, *MFA2 n*=2 biological replicates. (D) A bar graph of *PGK1* and *MFA2* mRNA half-lives in the strains indicated above in log2 scale. The *PGK1* and *MFA2* mRNA half-lives were normalized to the wild type or *edc3*Δ *lsm4*Δ*C* strain, respectively. Statistically significant pairings according to a two-tailed *t*-test indicated with their *P* values. Error bars indicate standard deviation.

We determined the relative fold-change of the deadenylation mutant compared with the corresponding background (i.e. half-life in the *ccr4*Δ *edc3*Δ *lsm4*Δ*C* strain compared with the *edc3*Δ *lsm4*Δ*C* mutant). The *ccr4*Δ mutant exhibited a 1.6- and 3-fold increase in the stability of the *PGK1* and *MFA2* mRNA, respectively (Fig. 3A,D). These results provide two insights into the mechanism of mRNA degradation: first, they suggest that the *MFA2* mRNA deadenylates faster than the *PGK1* mRNA, as reported previously (Tucker et al., 2001). Second, *PGK1* was significantly threefold more stable in the *ccr4*Δ *edc3*Δ *lsm4*Δ*C* mutant than in the wild type, a result consistent with faster deadenylation in this strain (Fig. 3D); however, the fold stabilization for the *MFA2* mRNA in the *ccr4*Δ mutant was comparable to that of the wild type. We attribute this result to the rapid deadenylation of the *MFA2* mRNA (Decker and Parker, 1993), which may not be able to be significantly accelerated further in the *edc3*Δ *lsm4*Δ*C* mutant.

The stabilization provided by the *ski2*Δ mutant was reduced in the *edc3*Δ *lsm4*Δ*C* mutant for the *MFA2* mRNA. This result supports a model in which the degradation of full-length mRNA by the exosome is reduced in the *edc3*Δ *lsm4*Δ*C* mutant background. Concomitantly, there is an increased reliance on decapping-dependent degradation (Fig. 3C,D). Because the decay intermediate is primarily degraded by the cytoplasmic exosome, it is more stable and prominent in the *ski2*Δ mutant (indicated by an arrow labeled D.I. in Fig. 3C,D).

Similarly, in the *xrn1* deletion mutant, which is deficient in decapping-dependent mRNA degradation (Muhlrad et al., 1994), the *MFA2* and *PGK1* mRNAs had increased stabilization in the *xrn1*Δ *edc3*Δ *lsm4*Δ*C* mutant background (Fig. 3B,D). Because of the deletion of *XRN1*, the lower band was absent in the *MFA2* mRNA blot, thus indicating that the trapped mRNA decay intermediate was not formed. The results from the *xrn1* deletion were consistent with increased deadenylation of mRNA. In yeast, deadenylated mRNA is preferentially directed to the decapping-dependent pathway, especially for *MFA2* which specifically recruits decapping activators (Cao and Parker, 2003; Chowdhury et al., 2014; Sharif et al., 2013). The results for the *xrn1*Δ mutant therefore suggest that the relative contribution of mRNA decapping to decay may be increased. Together, these results suggest that the reduced mRNA stability in the *edc3*Δ *lsm4*Δ*C* mutant is attributable to increased deadenylation and increased targeting to the decapping-dependent decay pathway.

### The mRNA decay systems are equally active in both the wild-type and *edc3Δ lsm4ΔC* mutant

**Fig. 4.**
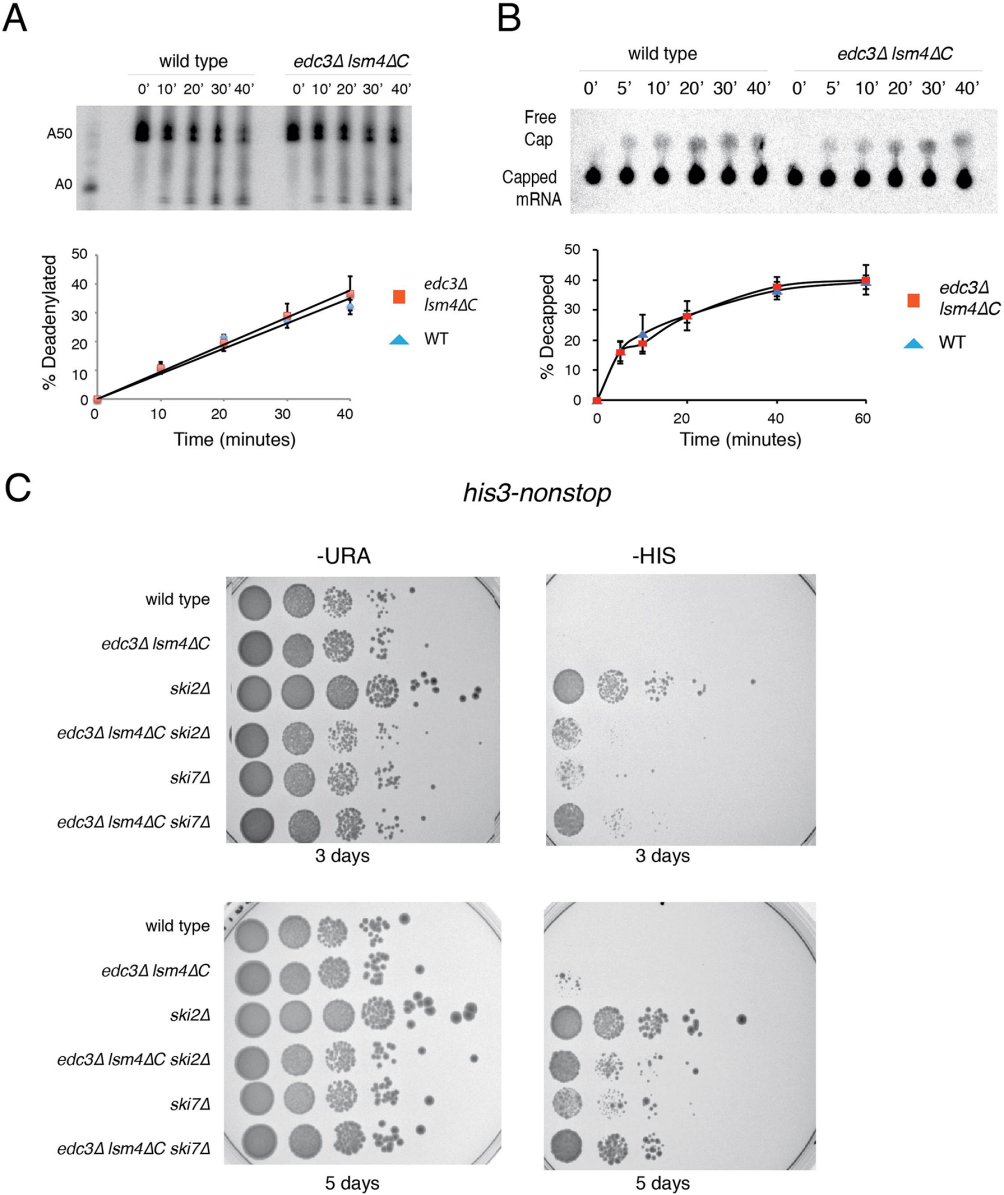
**The major mRNA decay systems have similar activities in the wild-type and *edc3*Δ *lsm4*Δ*C* strains.** (A) Deadenylation assay of transcribed *MFA2* 3′UTR with 50 3′ adenosines. Equal concentrations of purified TAP tagged Ccr4 from wild-type and *edc3*Δ*lsm4*Δ*C* cells were used. Error=s.d.; *n*=3 biological replicates. (B) Decapping assay of m7G cap labeled mRNA. Equal concentrations of purified TAP tagged Dcp1 from wild-type and *edc3*Δ*lsm4*Δ*C* cells were used. Error=s.d.; *n*=3 biological replicates. (C) Growth assay of yeast with a plasmid containing a non-stop variant of the *HIS3* gene in strains deleted for the *HIS3* gene as well as the gene indicated. Strains grown on synthetic complete plates lacking uracil or histidine respectively for both three and five days.

Our experimental evidence suggested that the more rapid decay observed in the *edc3*Δ *lsm4*Δ*C* mutant occurred via faster deadenylation by the Ccr4/Not deadenylation complex. One explanation for such an effect could be increased enzymatic activity. Using purified Ccr4 complexes from the wild-type and *edc3*Δ *lsm4*Δ*C* mutant strains, we observed no difference in the *in vitro* deadenylation rates (Fig. 4A). Because the dependence on the decapping pathway was significantly increased for *MFA2 in vivo* (Fig. 3D), we examined the *in vitro* mRNA decapping by using purified decapping complex (LaGrandeur and Parker, 1998). We observed similar decapping rates between the wild-type and *edc3*Δ *lsm4*Δ*C* mutants as assessed by the release of radiolabeled m7G cap from *in vitro* transcribed *MFA2* mRNA (Fig. 4B). Finally, we considered whether the cytoplasmic exosome activity might be altered between the strains. We performed an *in vivo* assay to examine exosome activity, using an mRNA lacking a stop codon. Such mRNAs are recognized and destroyed by the cytoplasmic exosome (van Hoof et al., 2002). Active mRNA destruction by the exosome is assessed by growth on a medium lacking histidine using strains deleted for the *HIS3* gene, with only a plasmid borne *HIS3* lacking a stop codon (non-stop *HIS3*). Strains defective in exosome activity should not be able to grow on medium without added histidine, as observed for the *ski2* and *ski7* mutants (Fig. 4C) (Schaeffer et al., 2008). The inabilities of the wild-type and *edc3*Δ *lsm4*Δ*C* mutant strains to grow on medium lacking histidine indicate that the cytoplasmic exosome is active in both strains. Our evaluations of the decapping, deadenylation and the cytoplasmic exosome systems suggested that all three systems are equally active in both strains.

### The *edc3Δ lsm4ΔC* mutant has alterations in mRNA decay protein levels, mRNA decapping enzyme localization and translation

**Fig. 5.**
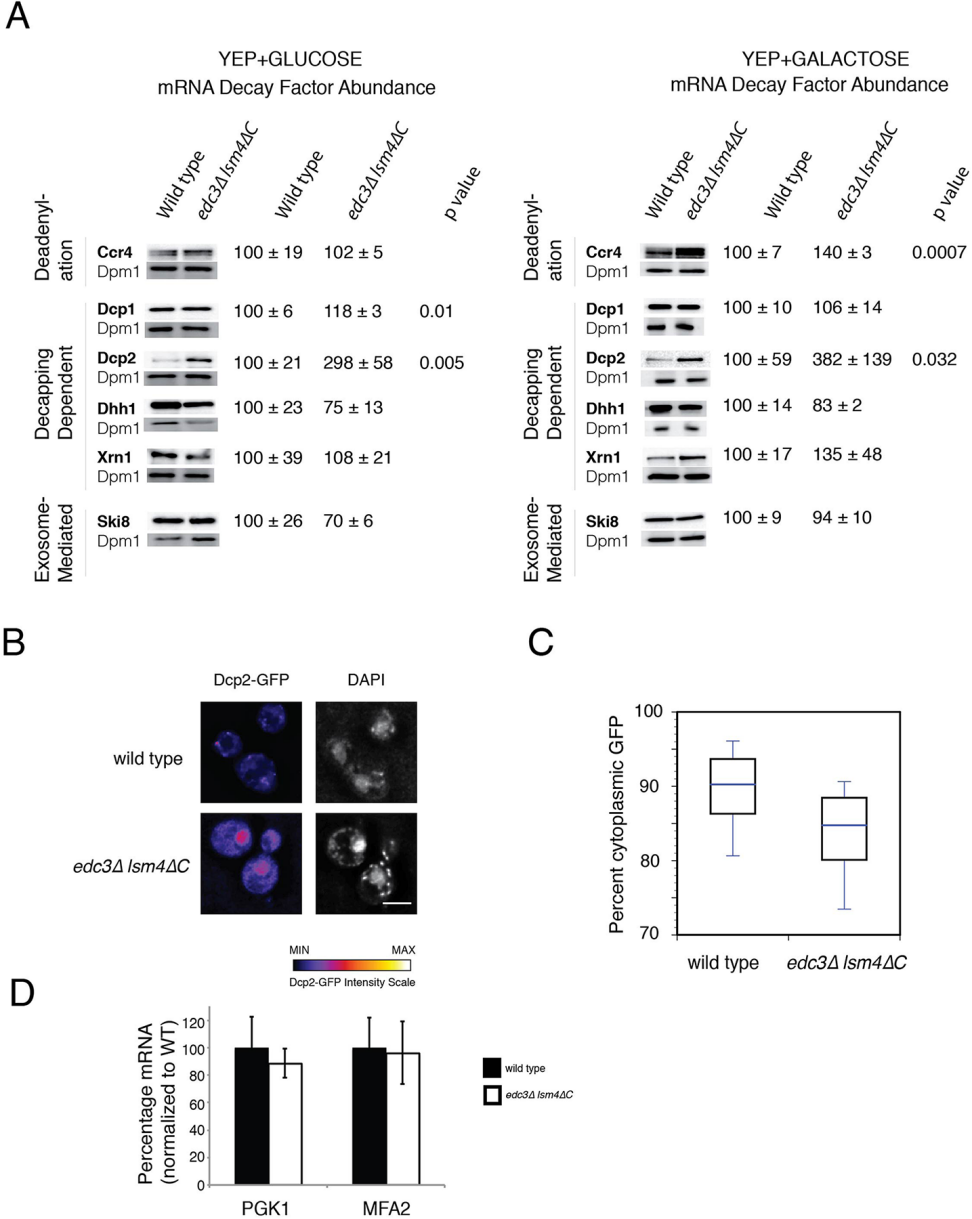
**Decay factor protein abundance, Dcp2 localization and mRNA levels in wild-type and *edc3*Δ *lsm4*Δ*C* mutant strains.** (A) Quantification of C-terminal TAP tagged mRNA decay factors protein levels in wild-type and *edc3*Δ *lsm4*Δ*C* strains. Cells grown in either glucose or galactose containing media. Loading normalized to the Dpm1 protein abundance. (B) Wild-type and mutant strains depicting Dcp2-GFP intensity in unstressed live cells. To the right is the DAPI staining. (C) Percentage of Dcp2-GFP that is cytoplasmic as determined from sum-projected Z-stacks, masked and intensity within the cytoplasm, box showing quantification of cytoplasmic Dcp2-GFP (line, median; box 25th and 75th percentiles; whiskers, 10th and 90th percentiles, *n*=50 biological replicates). (D) The relative full-length mRNA was determined for *MFA2* and *PGK1* in the strains indicated as compared to the *SCR1* loading control. The bars represent mean percentage of the indicated mRNA in the strain normalized to the WT background. Error bars indicate standard deviation, *n*=3 biological replicates. Significance determined in comparison to wild type by two-tailed *t*-test.

We observed a differential effect of the deadenylase and decapping complex mutants on mRNA degradation *in vivo* between the wild-type and *edc3*Δ *lsm4*Δ*C* strains (Fig. 3D). Because the activities of the decapping enzyme and deadenylase complex *in vitro* and cytoplasmic exosome *in vivo* were similar (Fig. 4), we examined the levels of key mRNA decay proteins between these strains. We found that most mRNA decay proteins examined were present at similar levels when the yeast was grown in galactose or glucose normalized to the ER membrane protein Dpm1 (Fig. 5A). However, in the *edc3*Δ *lsm4*Δ*C* strain, Dcp2, the enzymatic subunit of the decapping enzyme, was significantly more abundant when cells were grown in either carbon source. The levels of the cytoplasmic deadenylase Ccr4 and the non-catalytic subunit of the decapping enzyme Dcp1 were elevated to a significant extent when cells were grown in galactose or glucose respectively (Fig. 5A).

Whereas Dcp2 was elevated in the *edc3*Δ *lsm4*Δ*C* strain, we also observed that Dcp2 was concentrated in the nucleus in this mutant (Fig. 5C). Previous studies show a similar Dcp2 nuclear accumulation in the *edc3*Δ *lsm4*Δ*C* mutant, although this accumulation was not remarked upon (Decker et al., 2007; Grousl et al., 2009). We found that cytoplasmic Dcp2 was significantly reduced in the *edc3*Δ *lsm4*Δ*C* strain (*P*<1×10^−9^) by nuclear co-localization using DAPI (Fig. 5C). The greater nuclear accumulation may alter the effect of the elevated levels of Dcp2. Consistent with this model, we observed a similar level of *MFA2* and *PGK1* mRNA, normalized to *SCR1* RNA, in the wild-type and *edc3*Δ *lsm4*Δ*C* mutants (Fig. 5D).

**Fig. 6.**
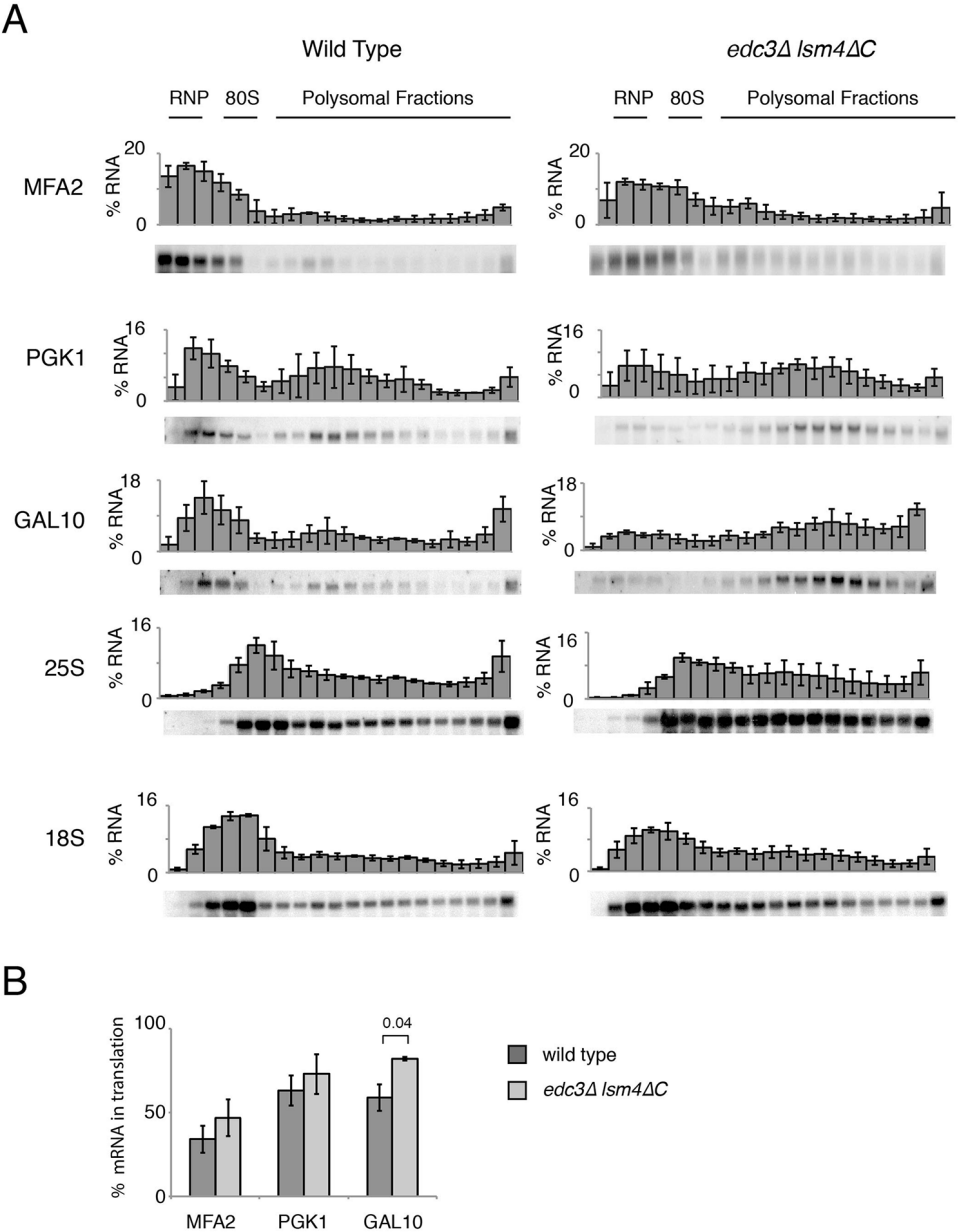
**Sucrose gradient distribution of mRNA and ribosomal subunits for wild-type and the *edc3*Δ *lsm4*Δ*C* strains.** (A) Northern blot analysis of fractions from a sucrose gradient after ultracentrifugation for the wild-type and *edc3*Δ *lsm4*Δ*C* strains. Each panel indicates the mRNA probed, a representative northern and quantification of intensity in each fraction (error bars indicate standard deviation, *n*=3 biological replicates). Yeast grown in standard yeast extract/peptone medium (YEP) supplemented with 2% galactose. (B) Quantification of the percentage of the indicated mRNAs in the translating fractions (80S and polysomal). Error bars indicate standard deviation. Significant P value is indicated as determined by *t*-test.

An alternative source of altered mRNA stability can come from changes in the translation of mRNAs (Drummond et al., 2011; Huch and Nissan, 2014; Jacobson and Peltz, 1996). We therefore examined the polysome profiles of extracts from yeast exponentially growing in glucose to gain insight into their translation. We found no differences between the *edc3*Δ *lsm4*Δ*C* strain, the wild type and the *edc3*Δ and *lsm4*Δ*C* mutants (data not shown), as reported previously (Decker et al., 2007). Because there were no gross alterations in translation as assessed by polysome analysis, we used northern blotting to examine the distribution of mRNAs separated on a sucrose gradient. We probed for the *PGK1*, *MFA2* and *GAL10* mRNAs after induction by growth in galactose for the wild-type and *edc3*Δ *lsm4*Δ*C* strains (Fig. 6A). In summary, we found a larger portion of these mRNAs in actively translating sucrose gradient fractions in the *edc3*Δ*lsm4*Δ*C* mutant than in the wild type (Fig. 6B). These fractions are presumably associated with ribosomes and therefore are not localized in P bodies (Parker and Sheth, 2007).

### Deadenylation and decapping dependent growth in the *edc3Δ lsm4ΔC* mutant

**Fig. 7.**
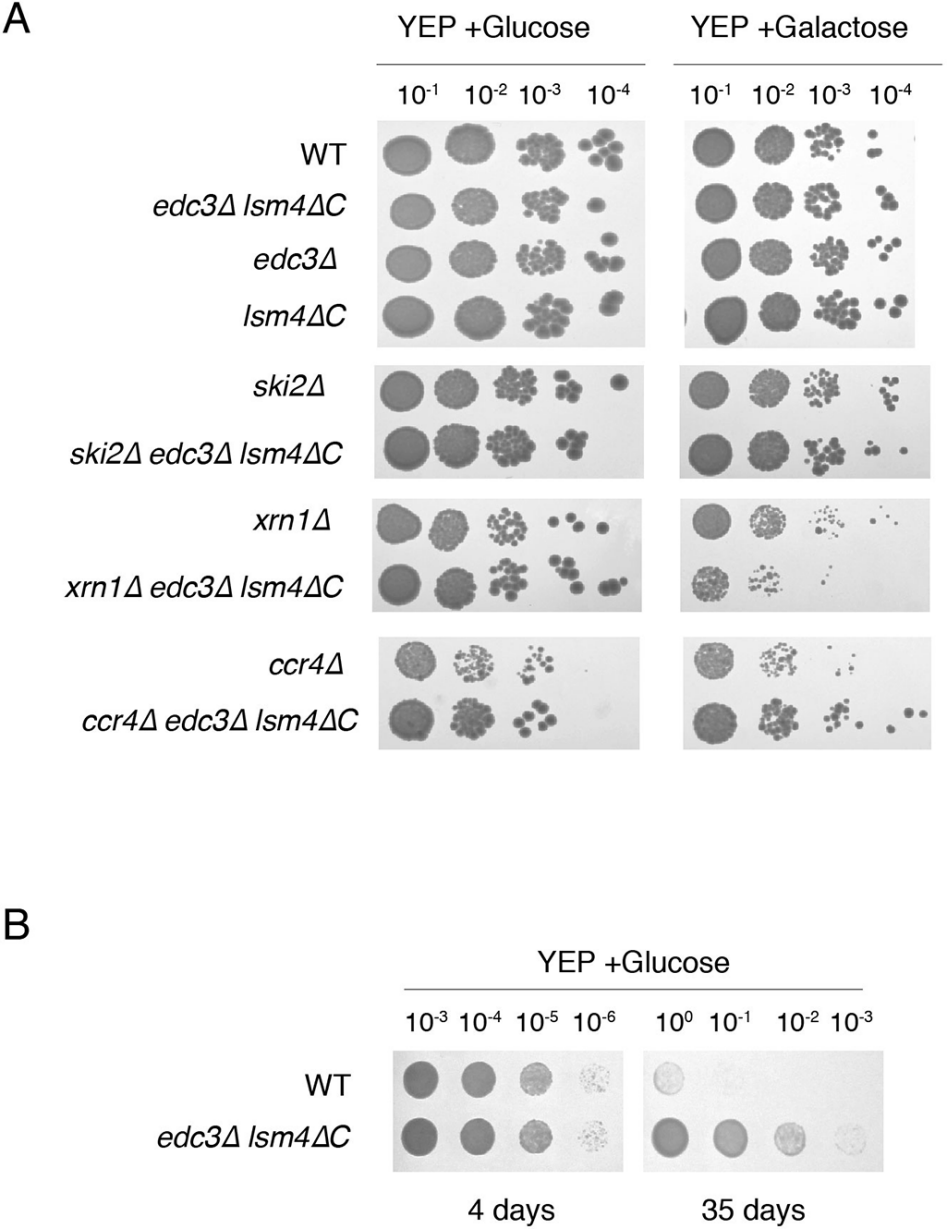
**The *edc3*Δ *lsm4*Δ*C* strain has altered growth in decay mutants and increased long-term survival viability.** (A) Serial dilutions (1:10) of wild type (WT) and the *edc3*Δ *lsm4*Δ*C* mutant strain grown on YEP with glucose or galactose for carbon source as indicated (*n*=3 biological replicates). (B) Wild-type and *edc3Δ lsm4ΔC* mutant yeast grown in liquid YEP+2% glucose and plated on YEP+2% glucose plates after 4 or 35 days (*n*=3 biological replicates).

Because the mRNA degradation pathway and mRNA stability was altered for the *edc3*Δ *lsm4*Δ*C* mutant, we asked whether the growth might be affected for cells in which mRNA transcription was increased. We therefore examined whether P bodies would be important for adaptation to higher mRNA levels due to galactose induction (Lavut and Raveh, 2012). The growth rate was largely similar in the wild-type, *edc3*Δ *lsm4*Δ*C* mutant and single mutation strains with galactose as the carbon source, albeit with a faster doubling rate in the *edc3*Δ *lsm4*Δ*C* mutant (Fig. 7, Fig. S1).

Despite the similar exponential growth in liquid culture, we postulated that the altered mechanism of mRNA decay might affect growth in the presence of elevated mRNA levels (Fig. 7, Fig. S1). We therefore examined decay pathway mutants expressing elevated mRNA levels in the wild-type and *edc3*Δ *lsm4*Δ*C* mutant strains and observed three patterns: first, the *ski2*Δ mutant did not show any difference in growth between the wild-type and *edc3*Δ *lsm4*Δ*C* mutant strain; therefore, there was no growth inhibition due to the overexpression of mRNA when the exosome was inactivated. Second, in the *xrn1*Δ mutant, the *edc3*Δ *lsm4*Δ*C* mutant was inhibited by the overexpression of mRNA and had tenfold less survival than that of the wild type. This finding suggests a greater dependence on the decapping-dependent pathway in the *edc3*Δ *lsm4*Δ*C* mutant, as indicated by our half-life data. Finally, in the *ccr4*Δ deadenylase mutant, the wild-type strain exhibited slower growth and a fivefold reduced survival rate.

If the mRNA destabilization in the *edc3*Δ *lsm4*Δ*C* mutant is related to P bodies, then these data suggest that P bodies stabilize mRNA. As such, they could also provide a competitive advantage to the cell. We therefore examined whether P bodies increased viability for yeast entering stationary phase. Surprisingly, we observed that yeast unable to form P bodies were more viable after extended incubation in stationary phase (Fig. 7B) suggesting that there may be a competitive cost to retaining the ability to form P bodies.

## DISCUSSION

In this work, we examined the effects of the *edc3*Δ *lsm4*Δ*C* mutant in mRNA metabolism. Edc3 and Lsm4 have been well established as components of the decapping complex and the decapping activating Lsm1-7 complex, respectively. Mutations in decapping factors result in increased mRNA stability. In contrast, we found that the *edc3*Δ and *lsm4*Δ*C* mutations individually behave as expected by either increasing or not affecting mRNA stability (Fig. 2C). However, when the two mutations were combined in the *edc3*Δ *lsm4*Δ*C* mutant, we found that the mutant exhibits reduced mRNA stability for mRNAs (Fig. 2C). By combining the *edc3*Δ *lsm4*Δ*C* mutant with mutations in mRNA decay pathways, we obtained insights into the more rapid decay observed; specifically, the *edc3*Δ *lsm4*Δ*C* mutant has increased dependence on deadenylation by the Ccr4/Not complex and decapping with a concomitant reduction in 3′-to-5′ degradation mediated by the exosome (Fig. 3D). These results suggest that the importance of deadenylation and decapping is increased in the *edc3*Δ *lsm4*Δ*C* mutant. Furthermore, we found that the enzymatic decay systems had similar activity (Fig. 4). These results suggest an alternative source for the altered mRNA decay.

Whereas most of the mRNAs examined here had shorter half-lives, we asked why the shorter-lived mRNAs were not significantly destabilized. A similar effect has also been observed in previous examinations of the role of P bodies in mRNA degradation (Decker et al., 2007; Eulalio et al., 2007; Stoecklin et al., 2006). These studies have examined short-lived mRNAs on the basis of the presumption that the absence of P bodies would stabilize mRNA. Detection of faster decay in unstable mRNA molecules may be precluded by a limit on the rate of deadenylation as well as by the difficulty of assessing the difference between very short half-lives. In the *edc3*Δ *lsm4*Δ*C* mutant, the decrease in mRNA stability, whatever the source, might have had the same limitation as that in previous studies.

Whereas we found the individual mutations *edc3*Δ and *lsm4*Δ*C* significantly stabilized or had a neutral effect on mRNAs. The combination of the two mutations resulted in destabilization. How could mutations in two decapping factors result in reduced mRNA stability? We can envision three possibilities.

The first possibility is that the altered levels of mRNA decay proteins directly promote the faster degradation observed. Specifically, we found that the levels of the catalytic subunit of the decapping enzyme (Dcp2) were elevated in the *edc3*Δ *lsm4*Δ*C* mutant under growth in glucose and galactose (Fig. 5A). In addition, Dcp1 was significantly elevated in abundance in the *edc3*Δ *lsm4*Δ*C* mutant when grown in glucose. Similarly, when grown in galactose, Ccr4 had significantly increased levels in the *edc3*Δ *lsm4*Δ*C* strain.

These decay factor levels might not cause the increase in degradation observed in the *edc3*Δ *lsm4*Δ*C* mutant. For example, efficient Dcp2 activity depends on the presence of Dcp1 in yeast, which was not concomitantly increased when grown in galactose (Beelman et al., 1996; Floor et al., 2010; She et al., 2004; Tharun et al., 2000). Since we observe increased degradation in the *edc3*Δ *lsm4*Δ*C* mutant whether the cells are grown in galactose or glucose, the Ccr4 and Dcp1 levels, which are only elevated in specific carbon sources, also may not be the cause of faster degradation (Fig. 2C). However, we can not exclude that these elevated levels have some effect. For instance, overexpression of Ccr4 on a high copy plasmid has been reported to increase the decay rate of PGK1, but not MFA2 (Tucker et al., 2002), and elevated Dcp1 would allow the elevated Dcp2 to have greater activity. Finally, we found that much of the additional Dcp2 was located in the nucleus (Fig. 5B,C). At least three lines of evidence suggest that the excess nuclear Dcp2 protein might not be expected to decap mRNA. First the poly(A) tails of the nuclear-retained mRNA are longer than those of cytoplasmic mRNA, and this phenomenon has been reported to result in decapping inhibition (Decker and Parker, 1993; Hilleren and Parker, 2001; Jensen et al., 2001). Second, nuclear degradation of normal mRNA is slower than cytoplasmic decay and proportional to time spent in the nucleus, in which mRNAs are exported within seconds in yeast (Das et al., 2003; Kufel et al., 2004; Oeffinger and Zenklusen, 2012). Third, 5′-to-3′ nuclear degradation is carried out by Rat1 rather than Xrn1 (Bousquet-Antonelli et al., 2000; Das et al., 2003). We observed a substantially increased dependence on Xrn1 in the mutant defective in P body formation, a result consistent with cytoplasmic degradation (Fig. 3D). We cannot preclude the possibility that altered decay factor levels account for the reduced mRNA stability in *edc3*Δ *lsm4*Δ*C* mutant; however, these results, together with results from previous studies, suggest that the altered levels may not be the origin of the faster degradation observed in the *edc3*Δ *lsm4*Δ*C* mutant.

A second possibility for the reduced stability in the *edc3*Δ *lsm4*Δ*C* mutant could be a rearrangement of RNPs on mRNAs, perhaps through altered decay protein levels, as discussed above, or through the absence of both Edc3 and the glutamine/asparagine rich domain of Lsm4. Both Edc3 and Lsm4 have previously been shown to be involved in enhanced mRNA decay and decapping both *in vivo* and *in vitro* (Badis et al., 2004; Dong et al., 2007; Fromm et al., 2012; Harigaya et al., 2010; Kshirsagar and Parker, 2004; Nissan et al., 2010; Tharun et al., 2000). Although both individually promote mRNA decapping, it is possible that they could act in an antagonistic manner when both are absent, as suggested by the increased rate of decay observed only when the mutations were combined (Fig. 2C). This mechanism might be possible in the absence of some of the positive activators that bind to Dcp2 and normally promote both general and specific mRNA degradation. These findings suggest the possibility of a combinatorial code for mRNA decapping (He and Jacobson, 2015).

A third potential source for the reduced mRNA stability observed in the *edc3*Δ *lsm4*Δ*C* mutant could be linked to its inability to function in the assembly of P bodies (Decker et al., 2007). Whereas all cytoplasmic mRNAs are ultimately subject to destruction, in this hypothesis, mRNAs not bound in P bodies have a different mechanism of decay and decay more rapidly in their absence. P bodies have several roles in mRNA degradation by differentially acting on cytosolic, polysomal and P body bound mRNAs. Specifically, P bodies may sequester the mRNA decapping-dependent decay and deadenylation factors and alter the mechanism of mRNA degradation in the cell. In agreement with such a model, recent evidence supports the notion that mRNA degradation primarily occurs on translating mRNA (Hu et al., 2009; Pelechano et al., 2015). Non-P body bound mRNA may be degraded by a decay mechanism more similar to that for ribosome associated mRNA. P body associated mRNA may degrade differently.

A plausible model for the reduced mRNA stability in the *edc3*Δ *lsm4*Δ*C* mutant in the P body assembly can be constructed on the basis of multiple recent studies on the effect of mRNA poly(A) length on P bodies and translation. First, mRNAs with long poly(A) tails are engaged in translation and are not found in P bodies under non-stress conditions, as used in this study (Aizer et al., 2014; Brengues and Parker, 2007; Hoyle et al., 2007; Zheng et al., 2008). Second, deadenylated mRNA with short poly(A) tails persist until they are either decapped or degraded from their 3′ end (Hu et al., 2009; Muhlrad et al., 1995). Third, these deadenylated mRNAs have reduced translation and accumulate in P bodies (Brengues and Parker, 2007; Hoyle et al., 2007; Jacobson and Peltz, 1996; Zheng et al., 2008). Finally, deadenylated mRNAs have much longer half-lives than poly(A)+ mRNAs, thus suggesting that the longest portion of their lifespan is P body associated (Presnyak et al., 2015). Together, these results are consistent with both cytosolic- and ribosomal-associated mRNAs having similar access to decay factors as well as P bodies having a fundamentally different environment. That is, the mRNA decay factors involved in decapping and deadenylation accumulate in P bodies, while excluding the exosome (Jain and Parker, 2013). The accumulation of deadenylation factors within P bodies may limit the rate of deadenylation of ribosome-associated and non-P-body-associated mRNAs (Cougot et al., 2004; Teixeira and Parker, 2007; Zheng et al., 2008).

Finally, we found that Edc3 and the glutamine/asparagine rich region of Lsm4 are important when combined with other deletions in mRNA degradation factors. For example, different decay pathways are required for enhanced growth in in the *edc3*Δ *lsm4*Δ*C* mutant and wild-type yeast after induction of elevated transcription (Fig. 7A). Interestingly, we observed increased long-term survival in the *edc3*Δ *lsm4*Δ*C* mutant compared with wild-type (Fig. 7B). Another study found the opposite result for long-term survival by using a mutant defective in P body formation (*pat1*Δ) (Lavut and Raveh, 2012; Shah et al., 2013). However, this result is complicated because Pat1 strongly affects mRNA stability genome-wide in yeast (Sun et al., 2013). Although we found that long-term survival was compromised in the *edc3*Δ *lsm4*Δ*C* mutant, a previous study has demonstrated that Edc3 and glutamine/asparagine rich region of Lsm4 is important for mating in yeast (Aronov et al., 2015). These data suggest that Edc3 and the glutamine/asparagine rich region of Lsm4, and thus potentially P bodies, could be evolutionarily selected for their importance in mating, despite compromising the long-term survival ability of yeast.

## MATERIALS AND METHODS

### Yeast strains and growth conditions

The genotypes of all strains used in this study are listed in Table S1. Strains were grown on either yeast extract/peptone (YP) medium or synthetic complete (SC) medium lacking amino acids, as indicated. As the carbon source, the media contained 2% galactose for glucose transcriptional shut-off experiments, otherwise 2% glucose was used. Strains were grown at 30°C. Where indicated, transcription was halted by the addition of thiolutin (6 µg ml^−1^). The thiolutin concentration was titrated to be the minimum level to inhibit transcription (Pelechano and Pérez-Ortín, 2008). Yeast genomic knock-out strains were generated using homologous recombination with regions of homology approximately 50 nucleotides upstream of the ATG and 50 downstream of the stop codon, in which the gene of interest was replaced with the nourseothricin *(natNT2)* and hygromycin B (*hphMX4*) antibiotic resistance genes as previously described (Janke et al., 2004). The knockout strains were confirmed by PCR and sequencing The plasmids used in this study are indicated in Table S3.

### Western blot analysis

TAP tags were detected by western blotting using rabbit Peroxidase-Anti-Peroxidase (Sigma) at 1:2500 (Lot #103M4822). Dpm1 was detected using mouse anti-Dpm1 (Life Technologies) at 1:2500 (Lot#1219807) and rabbit anti-mouse HRP (Agrisera, Vännäs, Sweden) at 1:2500 (Lot#1402).

### Microscopy

Live yeast cells were resuspended in appropriate minimal medium and visualized on a DeltaVision Spectris microscope with an Olympus 60×1.4NA objective without binning. Microscopic images were deconvolved using the classical maximum likelihood estimation algorithm in Huygens Essential 4.4 (SVI, Hilversum, The Netherlands). Each of the resulting images was depicted by means of sum intensity projections of a Z series of 20 slices of 0.25 µm thickness displayed with Fiji (Schindelin et al., 2012). Images in each panel are within the same contrast range displayed using the Fire lookup table in Fiji. Quantification of nuclear and cytoplasmic fluorescence was accomplished with sum projected Z-stacks of GFP and DAPI channels of live cells. The DAPI channel was thresholded using the Huang method (Huang and Wang, 1995). Approximately 100 cells were counted for wild-type and the *edc3*Δ *lsm4*Δ*C* mutant after removal of outliers in the DAPI channel. The intensities of the fluorescence were quantified with reference to the non-thresholded sum projection.

### Affinity protein purification

Two to six liters of yeast containing an integrated C terminal TAP tag attached to the protein of interest was grown to mid-log phase at 30°C in YPD medium. Cells were harvested and lysed using a pressure cell homogenizer (Stanstead Fluid Power). Cells were purified as previously described (Puig et al., 2001). Briefly, proteins were affinity-purified from cleared cell lysate using IgG resin (GE Healthcare), eluted with AcTEV (Invitrogen) and dialyzed into lysis buffer containing 50% glycerol for use in enzymatic assays.

### Decapping assay

The decapping enzyme was purified from the wild-type and *edc3*∆ *lsm4*∆*C* mutant strains by protein A purification of the C-terminal tagged Dcp1 protein (yTN259 and yTN263). After purification as previously described (Puig et al., 2001), extracts were dialyzed overnight into lysis buffer with 50% glycerol. The protein concentrations used in the assay were normalized by western blot analysis. Capped mRNA was produced as previously described (LaGrandeur and Parker, 1998). After T7 transcription of the XbaI-linearized pTN226 plasmid, mRNA was capped with the Vaccinia Capping System (New England Biolabs) with radiolabeled GTP. Labeled mRNA was separated on a urea polyacrylamide gel and the full length mRNA was identified by phosphorimager; the band was cut out and eluted overnight. Decapping was performed as previously described (LaGrandeur and Parker, 1998). Briefly, the decapping reaction contained cap-radiolabeled mRNA, purified decapping enzyme from the wild-type and mutant strains, and 5 fmol m^7^G[^32^P]pppMFA2pG mRNA. The reaction was carried out in Mg buffer (50 mM Tris, pH 7.6, 5 mM MgCl_2_, 50 mM NH_4_Cl, 1 mM DTT). The released caps were monitored by PEI TLC plates developed in 0.75 M LiCl.

### Deadenylation assay

The deadenylation complex was purified from the wild-type and P body mutant strains by protein A purification of the C-terminal tagged Ccr4 protein (yTN282 and yTN296). After purification as previously described (Puig et al., 2001), extracts were dialyzed overnight into lysis buffer with 50% glycerol. The protein concentrations used in the assay was normalized by western blot analysis. Deadenylation was performed on short mRNA containing the final 50 nucleotides of the MFA2pG 3′ UTR and 50 nucleotide poly(A) tail. Briefly, pTN229 was linearized by HindIII and mung bean nuclease, and then transcribed from the T7 promoter in the presence of radiolabeled nucleotides (Tucker et al., 2002). The deadenylation assay was performed similarly to previously published protocols (Tucker et al., 2001; Wilusz et al., 2001). The reaction was performed in TW buffer (20 mM HEPES, pH 7, 50 mM KCl, 1 mM MgOAc and 1 mM DTT) with approximately 10 µM RNA. The reaction was started by the addition of purified Ccr4 complex and incubated at 30°C. The reaction was quenched with a stop solution (20 mM EDTA and 300 mM NaOAc).

### RNA analysis

RNA was purified by lysing yeast cells with glass beads, and this was followed by a phenol/chloroform/isoamyl alcohol extraction and ethanol precipitation. Northern blots were probed with the oligonucleotides listed in Table S2. The mRNA half-lives were determined using transcriptional shut-off with 6 µg ml^−1^ thiolutin or by use of an inducible *GAL* promoter integrated into the genome, which controls the *MFA2*pG and *PGK1*pG mRNAs. The optimal thiolutin concentration was examined by titration and half-life determination (Pelechano and Pérez-Ortín, 2008). The transcription from the *GAL* promoter was repressed by the addition of 4% dextrose to the cell medium after washing as indicated. Quantification of bands on northern blots was performed with Quantity One (Bio-Rad). The mRNA half-lives were determined by the best linear fits of mRNA band intensities normalized to the *SCR1* loading control. The mRNA half-lives were subjected to two-tailed unpaired Student's *t*-tests. Statistical significance was determined with a *P*-value cut-off of 0.05.

### Polysomal fractionation of cellular extracts

Sucrose gradient analysis was performed as previously described (Huch et al., 2016). Cells were grown in YEP+2% galactose to an OD ∼0.5. Harvesting was performed by centrifugation at room temperature for 1 min at 3000 ***g***, and the cells were frozen in liquid nitrogen. Cells were lysed at 4°C using glass beads with incubation on ice for 5 min after 2 min pulses with a Disruptor Genie with 1× TN buffer (50 mM Tris-HCl, pH 7.4, 150 mM NaCl, 1 mM DTT) supplemented with a 10 mM ribonucleoside-vanadyl complex, 0.5 mg ml^−1^ heparin and complete EDTA Free Protease Inhibitor (Roche). Cycloheximide was not used during cell harvesting due to its ability to eliminate P bodies (Coller and Parker, 2005; Sheth and Parker, 2003). Cellular debris was cleared by centrifugation at 1500 ***g*** for 2 min. The resulting supernatant was loaded onto a 15-50% sucrose gradient with an 80% sucrose cushion. After ultracentrifugation at 39,000 rpm for 90 min, the extract was aliquoted and frozen on dry ice for later RNA extraction.
